# Chemical Composition and in Vitro and in Silico Larvicidal Activity of *Piper* spp. Essential Oils and Their Mixtures Against *Aedes aegypti* (Diptera: Culicidae)

**DOI:** 10.3390/plants15111704

**Published:** 2026-05-31

**Authors:** Anderson de Santana Botelho, Clenilma Marques Brandão, Lucas Gabriel Póvoas Silva, Carlos Alexandre Holanda, Eliza de Jesus Barros dos Santos, Mabrouk Horchani, Ravendra Kumar, Karyme do Socorro de Souza Vilhena, Marcilene Paiva da Silva, Mozaniel Santana de Oliveira, Eloisa Helena de Aguiar Andrade

**Affiliations:** 1 Postgraduate Program in Chemistry, Institute of Exact and Natural Sciences, Federal University of Pará, Augusto Corrêa Street, S/N, Guamá, Belém 66075-900, PA, Brazil; anderson.botelho@ueap.edu.br (A.d.S.B.); eloisa@museu-goeldi.br (E.H.d.A.A.); 2 Faculty of Chemistry, State University of Amapá, Pres. Vargas Avenue, 650, Central, Macapá 68901-258, AP, Brazil; 3 Laboratory of Microbiological Analysis, Department of Chemistry, Federal Institute of Maranhão, São Luís-Monte Castelo Campus, São Luís 65030-005, MA, Brazil; clenilma.brandao@ifma.edu.br (C.M.B.); lucaspovoas@acad.ifma.edu.br (L.G.P.S.); 4 Natural Sciences Degree Coordination, Federal University of Maranhão, Bom Jesus Campus, Imperatriz 65915-060, MA, Brazil; holanda@ufma.br; 5 Postgraduate Program in Biological Sciences, Concentration Area—Tropical Botany, Federal Rural University of the Amazon and Emílio Goeldi Museum of Pará, Av. Perimetral, 1901, Terra Firme, Belém 66077-830, PA, Brazil; elizasantos@museu-goeldi.br; 6 Laboratory of Heterocyclic Chemistry, Natural Products and Reactivity (LR11Es39), Medicinal Chemistry and Natural Products, Faculty of Sciences of Monastir, University of Monastir, Avenue of Environment, Monastir 5000, Tunisia; horchani.mabrouk@gmail.com; 7 Department of Chemistry, College of Basic Sciences and Humanities, G.B. Pant Universityof Agriculture and Technology, U.S. Nagar, Pantnagar 263145, Uttarakhand, India; ravichemistry.kumar@gmail.com; 8 Faculty of Chemistry, Institute of Exact and Natural Sciences, Federal University of Pará, Augusto Corrêa Street, S/N, Guamá, Belém 66075-900, PA, Brazil; karyme@ufpa.br; 9 Faculty of Chemical Engineering, State University of Amapá, Pres. Vargas Avenue, 650, Central, Macapá 68901-258, AP, Brazil; marcilene.silva@ueap.edu.br; 10 Postgraduate Program in Pharmaceutical Sciences, Institute of Health Sciences, Federal University of Pará, Belém 66075-900, PA, Brazil; 11 Adolpho Ducke Laboratory, Coordination of Botany, Emílio Goeldi Museum of Pará, Perimetral Avenue, 1901, Terra Firme, Belém 66077-830, PA, Brazil

**Keywords:** *Piper*, larvicidal, *Aedes aegypti*, dillapiole, essential oil

## Abstract

Controlling the arbovirus vector *Aedes aegypti* represents a growing public health challange, intensifying the search for alternatives to combat the mosquito. In this context, the present study aims to evaluate the larvicidal activity of essential oils from three *Piper* species and their mixtures, as well as their preliminary toxicity and in silico acetylcholinesterase (AChE) inhibitory potential. The essential oils and mixtures were characterized by GC–MS. The larvicidal activity test was performed against third-stage larvae, and a preliminary toxicity test was preformed against *Artemia salina*. The results showed that the oils had a high content of phenylpropanoids such as safrole, dillapiole, and eugenol, as well as their derivatives. The mixtures showed lower toxicity when compared to the pure oils. *P. aduncum* oil showed the highest larvicidal action (LC_50_ = 26.2 µg/mL), followed by *P. callosum* (LC_50_ = 53.2 µg/mL), while *P. divaricatum* had the lowest activity (LC_50_ = 123.8 µg/mL). Among the mixtures, the combination of *P. divaricatum* and *P. aduncum* stood out for its synergistic effect. Molecular docking analyses suggested that phytoconstituents interact favorably with AChE, supporting a neurotoxic mechanism associated with enzyme inhibition. Thus, *Piper* essential oils and mixtures are promising alternatives for the control of *A. aegypti*.

## 1. Introduction

The *Aedes aegypti* mosquito is the primary vector for important arboviruses, such as dengue, Zika, and chikungunya, which pose serious public health problems in countries with tropical and subtropical climates [1,2]. The high adaptability of this vector to the environment, coupled with its growing resistance to widely used synthetic insecticides, has made it difficult to effectively control its populations [3]. Therefore, it is necessary to search for effective and environmentally safe alternatives for vector control.

The use of natural plant-derived products stands out as a promising approach to control the vector, especially essential oils (EOs), which contain compounds with recognized insecticidal, larvicidal, repellent, and ovicidal activity [4]. In addition to their biological effectiveness, EOs are biodegradable and generally have low toxicity to humans and less environmental impact, making them attractive alternatives to conventional compounds [5,6].

Among the promising EOs for controlling *A. aegypti* are those of the genus *Piper* (Piperaceae), which contain bioactive compounds in their composition, including phenylpropanoids, monoterpenes, and sesquiterpenes that exhibit, among other activities, insecticidal and larvicidal action against the arbovirus vector [7,8,9]. These biological activities observed for *Piper* EOs are directly related to the presence of compounds such as dillapiole and eugenol, which are found in some of these species and have already been reported to be active against mosquitoes [10,11,12].

In this context, the present study aimed to evaluate the larvicidal activity of EOs extracted from the leaves of *Piper callosum* Ruiz & Pav., *Piper divaricatum* G. Mey., and *Piper aduncum* L., both as pure EOs and as mixtures against *A. aegypti* larvae, in addition to performing chemical characterization, preliminary toxicity testing against *Artemia salina* L., and in silico acetylcholinesterase (AChE) inhibitory potential analyses, aiming to identify EOs and combinations of EOs effective in combating the vector.

## 2. Results and Discussion

### 2.1. Chemical Composition

The essential oils of *P. callosum*, *P. divaricatum*, and *P. aduncum* extracted by hydrodistillation yielded 2.77%, 2.18%, and 2.56%, respectively, based on leaf dry weight. A total of 47 compounds were identified among the essential oils and mixtures (Table 1). Of these, 19 constituted the EO of *P. callosum*, 19 were identified in the EO of *P. divaricatum*, and 32 were detected in the EO of *P. aduncum*. The mixtures of EOs presented intermediate compositions between the compositions of each EO that constituted them, and maintained the presence of all the major compounds in the composition.

The main chemical class found in the EOs of the species evaluated was phenylpropanoids, with contents ranging from 54.92 to 75.50%, followed by sesquiterpene hydrocarbons (3.38–39.76%), monoterpene hydrocarbons (3.67–16.63%), oxygenated monoterpenes (0–6.48%), and oxygenated sesquiterpenes (1.47–3.77%). The high phenylpropanoid content is mainly associated with the presence of safrole, eugenol, methyl eugenol, eugenol acetate, and dillapiole, known for their wide range of biological activities and their previously reported presence in the species studied [7].

In the EO of *P. callosum*, the major compounds were the phenylpropanoids safrole (59.42%) and methyl eugenol (12.56%), as well as the monoterpene hydrocarbon β-pinene (8.85%). Safrole and methyl eugenol are compounds with insecticidal activity, but they also have other reported activities such as antimicrobial, cytotoxic, anti-inflammatory, and analgesic activities [13,14]. As for β-pinene, this compound is widely found in various essential oils and has insecticidal and antifungal properties [15].

On the other hand, the EO of *P. divaricatum* presented a more diverse chemical profile of major compounds among the species studied, composed mainly of the phenylpropanoids eugenol (18.26%), eugenol acetate (18.02%), methyl eugenol (12.60%), and safrole (6.04%), as well as the sesquiterpene hydrocarbons β-elemene (12.96%), (*E*)-caryophyllene (9.57%), γ-muurolene (8.75%), and bicyclogermacrene (5.12%). The phenylpropanoids present in this oil are known for their insecticidal, analgesic, neuroprotective, anti-inflammatory, and antimicrobial activities [13,14,16], while sesquiterpenes have antitumor, antifungal, and larvicidal activities [17,18].

The EO of *P. aduncum* had as its main constituents the phenylpropanoid dillapiole (55.92%) and the monoterpene hydrocarbon (*E*)-β-ocimene (6.20%). Dillapiole is recognized for its insecticidal and larvicidal activities [19,20], and is often found in species with levels that vary depending on the place of origin [21]. (*E*)-β-ocimene, in turn, is a compound involved in plant defense mechanisms against herbivores [22].

The EO mixtures retained the main constituents of the source species, with variations in relative proportions depending on the combination of oils. In general, phenylpropanoids such as safrole, eugenol, methyl eugenol, eugenol acetate, and dillapiole were present in all mixtures. The combination of these compounds may result in synergistic effects, enhancing the activities of the isolated oils, as already observed in studies with dillapiole, the major compound of the EO of *P. aduncum* [20,21]. In addition, this combination can also broaden the spectrum of biological action and reduce the necessary concentration of each individual oil, which can minimize possible adverse effects. However, mixing these compounds can also generate antagonistic effects, reducing or nullifying the activity of bioactive compounds [23].

### 2.2. Preliminary Toxicity

**Figure 1:**
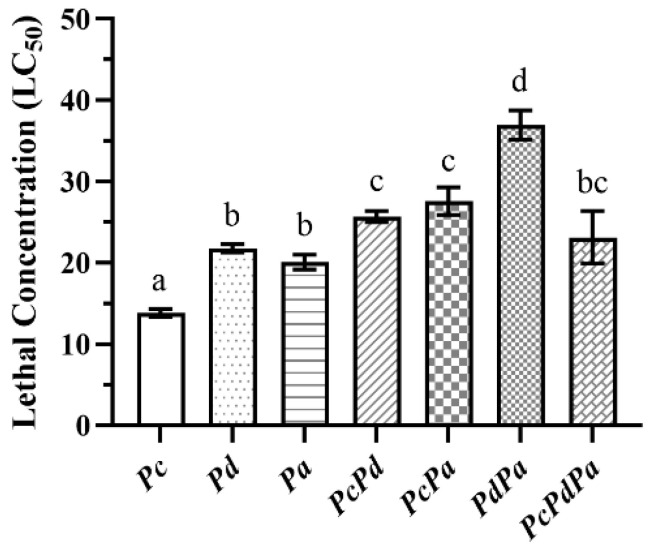
Comparative graph between the lethal concentrations (LC_50_) of *Piper* OEs and their mixtures against larvae of the microcrustacean *A. salina* after 24 h of exposure. Identical letters indicate that there were no statistically significant differences (*p* < 0.05), according to Tukey’s post hoc test.

The results of preliminary toxicity tests performed on *A. salina* larvae for the EOs of *P. callosum* (*Pc*), *P. divaricatum* (*Pd*), *P. aduncum* (*Pa*), and their mixtures are expressed as mortality at different concentrations in Table 2, and LC_50_ values are illustrated in Figure 1. All samples were toxic (LC_50_ < 1000 µg/mL) according to the parameter adopted by [24]. These results also reveal variations in toxicity among the tested essential oils and their mixtures, with significant differences (*p* < 0.05) between certain pairs of samples tested.

*Pc* exhibited the lowest LC_50_ value (13.9 ± 0.5 µg/mL), with statistically significant differences in relation to all samples tested, indicating that it was the most toxic. *Pd* and *Pa* exhibited intermediate toxicities with LC_50_ values of 20.1 ± 0.9 µg/mL and 21.8 ± 0.5 µg/mL, respectively, showing no statistically significant differences between them. These data suggest that the Eos of *P. divaricatum* and *P. aduncum* have lower toxicity levels than the EO of *P. callosum*, corroborating results reported in the literature [25,26].

When combined in binary and ternary mixtures, an increase in all LC_50_ values was observed, i.e., a reduction in the toxicity of these oils in mixtures compared to the pure oils. The mixtures *PcPd* (25.7 ± 0.7 µg/mL), *PcPa* (27.6 ± 1.7 µg/mL), and *PdPa* (37.0 ± 1.8 µg/mL), for example, exhibited lower toxicity than their isolated EOs, indicating a possible antagonistic effect among the constituents capable of reducing toxicity. Of these, the binary mixture *PdPa* (37.0 ± 1.8 µg/mL) was the least toxic, and the ternary mixture *PcPdPa* (23.2 ± 3.2 µg/mL) was the most toxic.

These preliminary toxicity results suggest that combining these EOs can considerably reduce the toxic effect, indicating a possible antagonistic effect among the constituents of the mixtures. Although there are currently no data in the literature on the antagonism of EO mixtures against *A. salina* larvae, this type of interaction has been observed in different biological activities and can be attributed to competition between compounds for the same biological action sites or to interactions that reduce the bioavailability of toxic components, among others [27,28,29].

### 2.3. Larvicidal Activity

**Figure 2:**
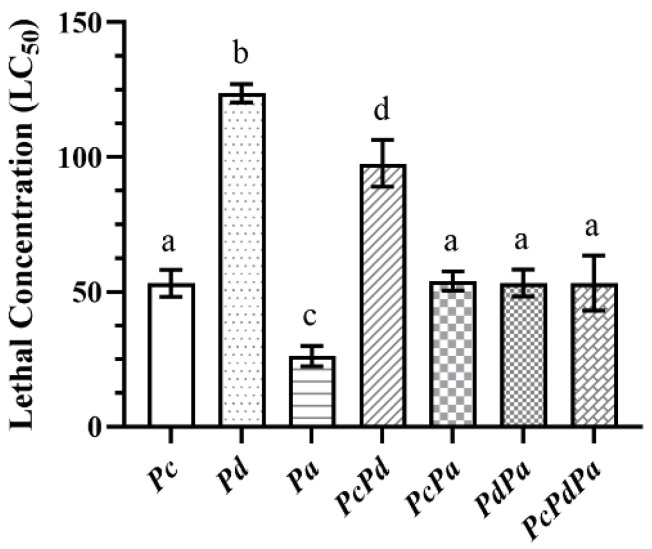
Comparative graph between the lethal concentrations (LC_50_) of *Piper* EOs and mixtures against *A. aegypti* larvae in the 3º stage after 24 h of exposure. Identical letters indicate that there were no statistically significant differences (*p* < 0.05), according to Tukey’s post hoc test.

**Figure 3:**
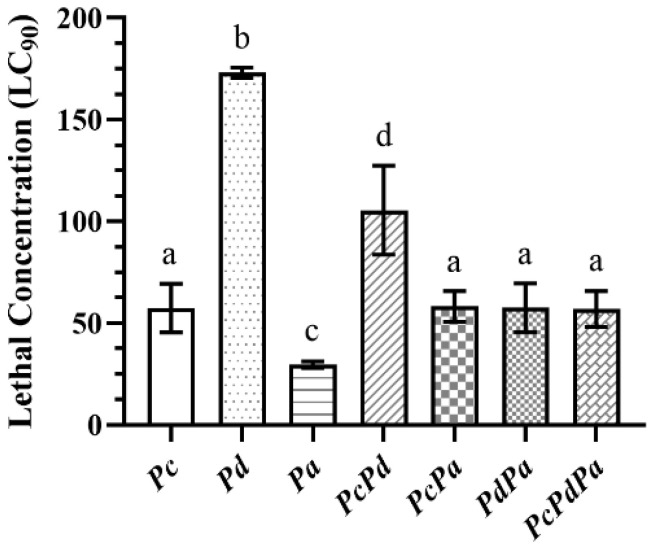
Comparative graph between the lethal concentrations (LC_90_) of *Piper* EOs and mixtures against *A. aegypti* larvae in the 3º stage after 24 h of exposure. Identical letters indicate that there were no statistically significant differences (*p* < 0.05), according to Tukey’s post hoc test.

The larvicidal activity data of the EOs of *P. callosum* (*Pc*), *P. divaricatum* (*Pd*), *P. aduncum* (*Pa*), and their mixtures against third-instar larvae of *Aedes aegypti*, are expressed as mortality at different concentrations in Table 3 and LC_50_ and LC_90_ values are illustrated in Figure 2 and Figure 3. The results show significant variations in toxicity between individual oils and their combinations. The data indicates that there were significant differences (*p* < 0.05) among the oils *Pc*, *Pd*, *Pa*, and the mixture *PcPd*, but there was no difference between the oil *Pc* and the mixtures *PcPa*, *PdPa*, and *PcPdPa*.

Among the oils and mixtures evaluated, the EO of *P. aduncum* (*Pa*) had the lowest mean lethal concentration (LC_50_ = 26.2 ± 3.8 µg/mL) and was classified as highly active according to the criteria established by [4,30,31]. These data are consistent with those on the larvicidal activity of *P. aduncum* EO in a study conducted by [32]. This high activity can be attributed to the high content of dillapiole present in the oil (55.92%), a phenylpropanoid with recognized larvicidal action [19,20]. Although this compound was not predominant in the study conducted by [33], the results are similar to the larvicidal activity observed by [32], indicating the possibility that other compounds, such as (*E*)-β-ocimene, or synergistic interactions between them, may be responsible for the observed activity.

The EO of *P. callosum* (*Pc*) also demonstrated relevant action with an LC_50_ of 53.2 ± 5.0 µg/mL and was classified as active. The main constituent of this oil was safrole (59.42%), a compound with insecticidal and larvicidal activity that may have contributed significantly to the observed activity [34]. In addition to this, compounds such as methyl eugenol and β-pinene, which are mainly present in EOs with larvicidal action against *A. aegypti*, may also have contributed to the toxicity observed [35].

On the other hand, *P. divaricatum* (*Pd*) EO was the least effective among the oils and mixtures, with an LC_50_ of 123.8 ± 3.4 µg/mL, and it was classified as only effective. Although this oil contains bioactive compounds such as eugenol, eugenol acetate, and methyl eugenol [11,36], the lower proportions of these constituents may explain its reduced larvicidal activity compared to the other oils and mixtures evaluated.

The binary and ternary mixtures of the EOs showed intermediate activities, reflecting the combined influence of the chemical compositions. The *PdPa* mixture had an LC_50_ of 53.3 ± 5.0 µg/mL, similar to that observed for *Pc*, with activity higher than the average lethal concentrations obtained for the oils that make up the mixture. This result suggests a positive effect of adding *Pa* to *Pd*, reinforcing the potential synergistic effect between the compounds in each oil. Among the compounds, this activity may be linked to the synergism between dillapiole and methyl eugenol in the EOs of *P. aduncum* and *P. divaricatum*, which have already shown a strong synergistic effect for insecticidal activity [20].

On the other hand, this effect was not observed in the *PcPa* mixture (LC_50_ = 54.0 ± 3.5 µg/mL), maintaining activity similar tothat of *Pc*, which may indicate an antagonistic effect between the oils or dilution of active compounds. Meanwhile, the binary mixture *PcPd* and the ternary mixture *PcPdPa* showed average activities between the oils that compose them (with LC_50_ values of 97.7 ± 8.7 and 53.3 ± 10.3 µg/mL, respectively). These results demonstrate that combining EOs may be a promising strategy to optimize the larvicidal effect of oils and reduce toxicity, but further studies on their toxicity are needed to ensure the safety of their application.

### 2.4. Molecular Docking Investigations

#### 2.4.1. Docking Validation

**Figure 4:**
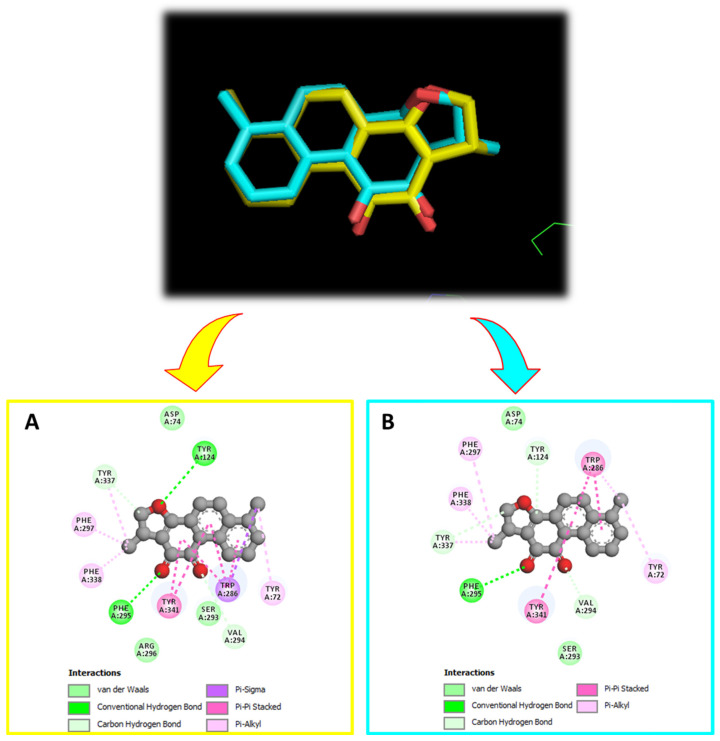
2D model of different interactions formed by the co-crystallised (yellow colour) (**A**) and the re-docked native ligand (cyan colour) (**B**) within the active site of acetylcholinesterase (pdb: 4m0e).

Molecular docking investigations were performed to elucidate potential intermolecular interactions between the tested compounds and the selected receptor, aiming to clarify the biological relevance of these ligands. Indeed, to validate the docking protocol, the docked conformation of the native ligand (co-crystallized ligand) was superimposed onto the native ligand derived from the receptor’s crystal structure. The superposition (Figure 4) showed a very low root-mean-square deviation (RMSD) between the two conformations (shown in yellow and cyan). Based on the molecular docking results, the redocked co-crystallized ligand demonstrated favorable binding to the receptor (Figure 4B), reproducing interactions comparable to those observed in the original crystal structure (Figure 4A), thus confirming the precision and reliability of the docking protocol.

#### 2.4.2. Docking Outcomes Analyses

**Figure 5:**
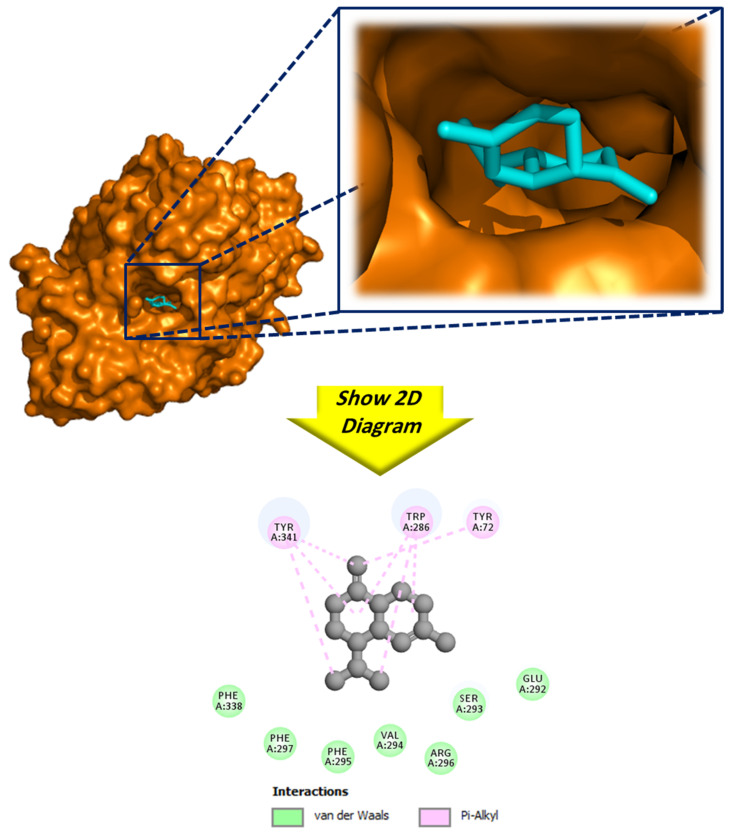
3D and 2D models of different interactions formed by the most bioactive compound γ-muurolene within the active site of acetylcholinesterase (pdb: 4m0e).

Against the acetylcholinesterase targeted enzyme, the results illustrated in Table 4 show that the majority of docked phytocompounds had docking scores ranging from −5.1 to −7.2 kcal/mol. Indeed, the most effective ligand was γ-muurolene, which exhibited the best docking score (binding energy value = −7.2 kcal/mol). The 3D plot in Figure 5 shows that γ-muurolene fits well in the binding cavity of the targeted enzyme AChE through the formation of many hydrophobic intermolecular contacts, such as Pi–Alkyls interactions, with the amino acid sequence: Tyr72, Trp286, and Tyr341.

**Figure 6:**
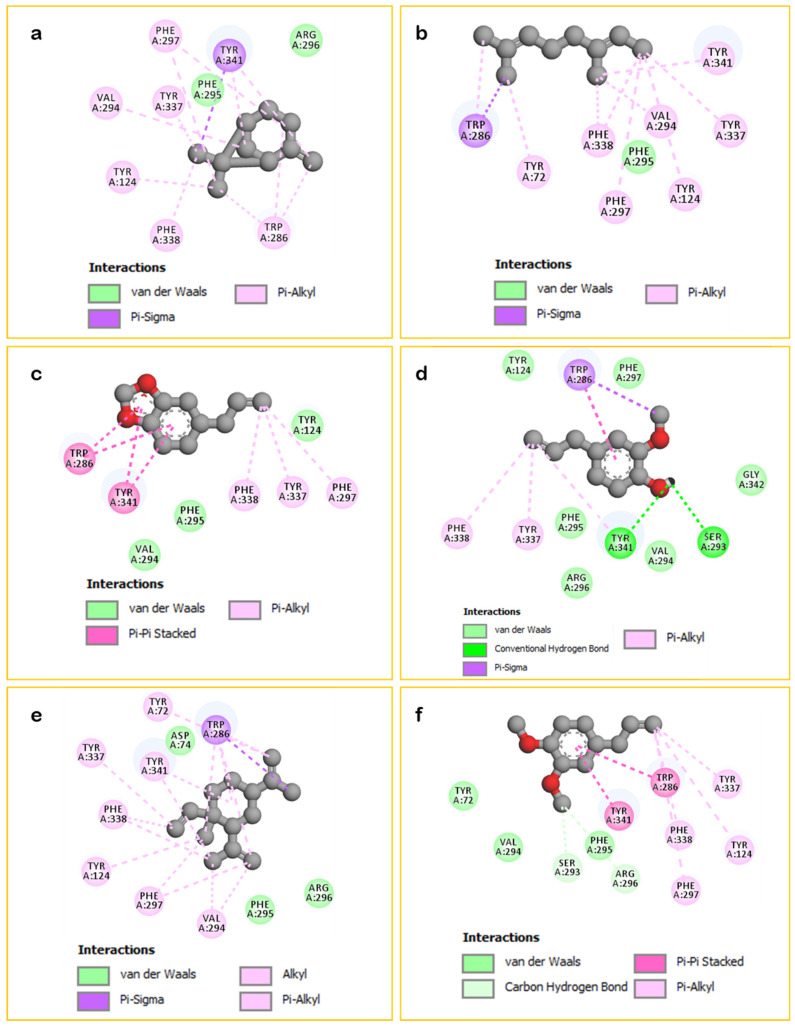
2D model of different interactions formed by the docked compounds. (**a**): β-pinene, (**b**): (*E*)-β-ocimene, (**c**): safrole, (**d**): eugenol, (**e**): β-elemene and (**f**): methyl eugenol within the active site of acetylcholinesterase (pdb: 4m0e).

**Figure 7:**
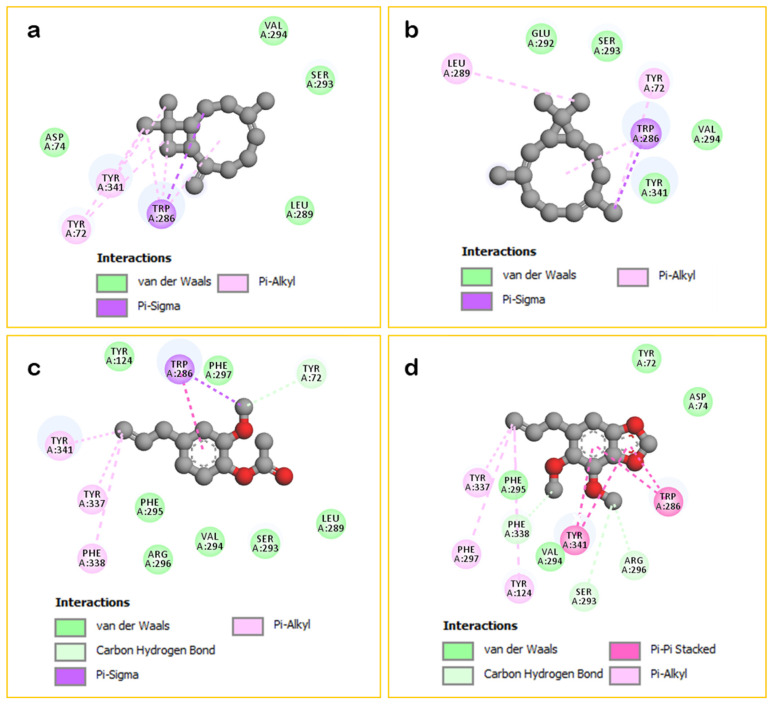
2D model of different interactions formed by the docked compounds. (**a**): (*E*)-caryophyllene, (**b**): bicyclogermacrene, (**c**): eugenol acetate and (**d**): dillapiole within the active site of acetylcholinesterase (pdb: 4m0e).

All other docked phytomolecules were involved in several interesting intermolecular contacts, as detailed in the 2D plots in Figure 6 and Figure 7. For more details, (*E*)-caryophyllene was found to be the second most bioactive ligand by displaying strong binding to the AChE’s active site with a Pi–Sigma interaction with Trp286 in addition to Pi–Alkyl interactions similar to those observed for its analogue γ-muurolene. Meanwhile, the third most effective ligand, β-elemene, was involved in a Pi–Sigma interaction with Trp286 in addition to many Alkyl and Pi-Alkyl contacts with Tyr72, Tyr124, Trp286, Val294, Phe297, Tyr377, Phe388, and Tyr341.

#### 2.4.3. Predictive ADME Analysis

ADME (absorption, distribution, metabolism, and excretion) data of the selected phytocompounds have been estimated. The predicted descriptors, including their pharmacokinetic and drug-likeness properties, are shown in the tabulated data. All tested ligands were found to correctly meet Lipinski’s rule and to share topological polar surface area (TPSA) values of 0.00 to 36.92 Å^2^, supporting the probability of predicted high passive oral absorption, as expressed by the consensus Log Po/*w* in the range of −5.93 to −4.65. Furthermore, the bioavailability score of 0.55 indicates higher bioactivity of the molecule.

As shown in Table 5, there were no P-glycoprotein (P–gp) substrates. This finding suggests good intestinal absorption and bioavailability of the compounds. Additionally, some tested ligands displayed low gastrointestinal absorption (GI). Almost all ligands were predicted to cross the blood–brain barrier (BBB), thus supporting their anti-AChE potentials. Furthermore, almost all tested compounds were found not to inhibit the main cytochrome (CYP 450) enzymes: CYP1A2, CYP2C19, CYP2C9, CYP2D6, and CYP3A4. Inhibition of these isoenzymes is certainly one of the major causes of drug-related pharmacokinetic interactions leading to toxic effects or other adverse effects.

**Figure 8:**
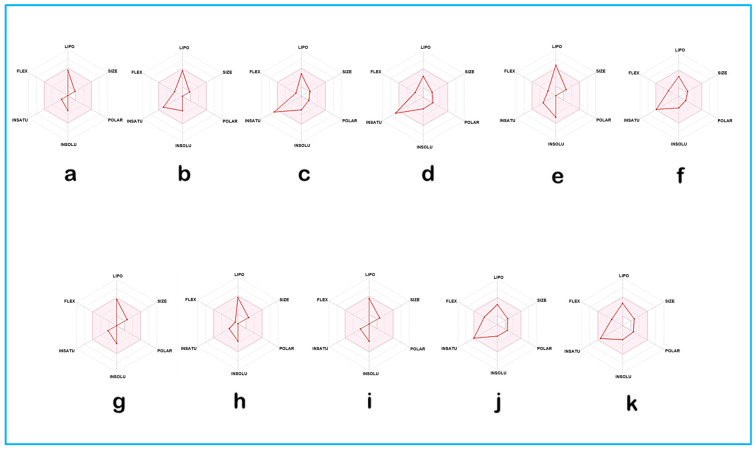
Bioavailability radar of selected phytoconstituants: (**a**): β-pinene, (**b**): (*E*)-β-ocimene, (**c**): safrole, (**d**): eugenol, (**e**): β-elemene, (**f**): methyl eugenol, (**g**): (*E*)-caryophyllene, (**h**): γ-muurolene, (**i**): bicyclogermacrene, (**j**): eugenol acetate, and (**k**): dillapiole.

**Figure 9:**
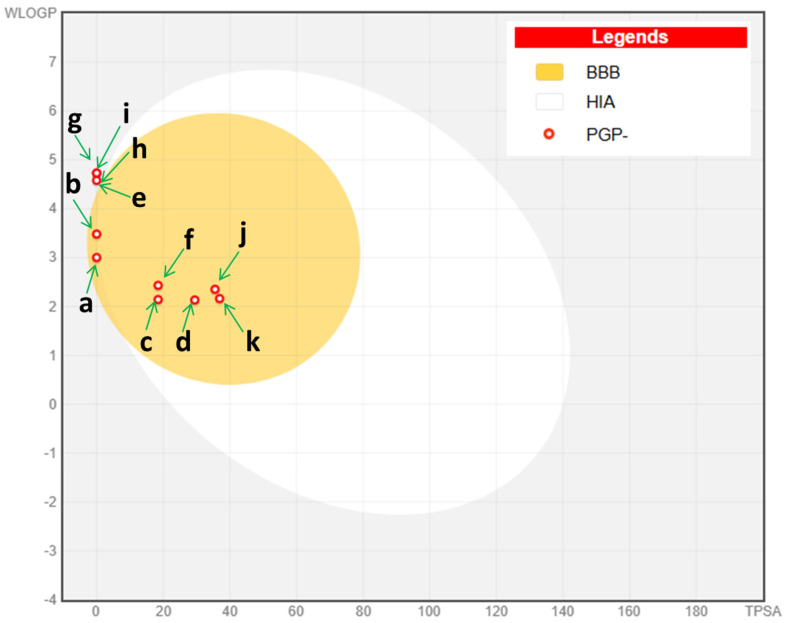
BOILED-EGG graph of the selected phytoconstituants: (a): β-pinene, (b): (*E*)-β-ocimene, (c): safrole, (d): eugenol, (e): β-elemene, (f): methyl eugenol, (g): (*E*)-caryophyllene, (h): γ-murolene, (i): bicyclogermacrene, (j): eugenol acetate, and (k): dillapiole.

The radar plot (Figure 8) shows that all tested molecules are within the pink zone, confirming their favorable drug-likeness and good bioavailability profile. In addition, Figure 9 presents the BOILED-EGG model, in which BBB penetration and GI absorption (HIA) of the substances can be predicted. This model distinguishes two regions: one corresponding to the GI absorption zone (HIA) and the other to BBB penetration (yolk). Neither “GI absorption” nor “BBB penetration” are indicated when a component is located in the gray zone. According to the obtained results, seven of the eleven phytoligands appear in the yellow (egg yolk) region, with red dots indicating a high probability of brain penetration (BBB) as non-substrates of P–gp.

## 3. Materials and Methods

### 3.1. Collection and Processing of Botanical Material

Samples of *P. callosum*, *P. divaricatum*, and *P. aduncum* were collected in the state of Pará, Brazil: *P. callosum* in the city of Abaetetuba (1°43′44.4″ S 48°52′04.5″ W) at 7:00 a.m. on 2 July 2023; *P. divaricatum* in Belém (1°46′45.5″ S 48°44′60.3″ W) at 11:00 a.m. on 13 November 2023; and *P. aduncum* in Belém (1°27′04.4″ S 48°26′45.2″ W) at 9:00 a.m. on 21 May 2024. The specimens were deposited in the Aromatic Plants collection of the João Murça Pires Herbarium, Museu Paraense Emílio Goeldi, Pará, Brazil, under voucher code MG184954 (*P. callosum*), voucher code MG165214 (*P. divaricatum*), and voucher code MG165196 (*P. aduncum*). After collection, the samples were dried in an air circulation oven at 35 °C until a constant weight was reached. The leaves were separated from the branches and ground using a knife mill.

### 3.2. Extraction of Essential Oils

The samples of dried and ground leaves were subjected to essential oil extraction by hydrodistillation using a modified Clevenger apparatus for 3 h. After extraction, the obtained essential oils (EOs) were centrifuged at 3000 rpm for 10 min, dehydrated with sodium sulfate, and centrifuged again under the same conditions. An aliquot (0.5 µL) of the EOs was diluted in 500 µL of hexane for chemical composition analysis by gas chromatography–mass spectrometry (GC–MS). The EOs were stored in amber glass vials and kept in a freezer at −17 °C for further analysis.

The yield of each EO was calculated on a moisture-free basis (MFB) as the ratio between the volume of oil obtained and the dry mass of the plant material used for extraction, considering the moisture content of the dried and ground leaves, which was determined using a Marte ID-50 moisture meter.

### 3.3. Preparation of EO Mixture

Three binary mixtures of the extracted essential oils (EOs) were prepared at a 1:1 ratio, consisting of *P. callosum*/*P. divaricatum* (*PcPd*), *P. callosum*/*P. aduncum* (*PcPa*), and *P. divaricatum*/*P. aduncum* (*PdPa*). A fourth ternary mixture was prepared at a 1:1:1 ratio consisting of *P. callosum*/*P. divaricatum*/*P. aduncum* (*PcPdPa*). These mixtures were characterized by GC-MS and subjected to preliminary toxicity and larvicidal tests.

### 3.4. Characterization of Essential Oils by GC-MS

The constituents of the EOs from the leaves of *Piper* species and mixtures were identified by gas chromatography coupled with mass spectrometry in a Nexis GC-2030 (Shimadzu, Kyoto, Japan) system equipped with a DB-5MS silica capillary column (30 m × 0.25 mm × 0.25 µm film thickness). Helium was used as the carrier gas at a linear velocity of 36.5 cm s^−1^, with the injector temperature at 250 °C, splitless injection, and oven temperature programming from 60 to 250 °C with a gradient of 3 °C/min. The mass spectrometer was operated in electron-impact mode at 70 eV, scanning the range from 39 to 500 da.s^−1^ with the ion source temperature at 220 °C. The retention index of all volatile constituents was calculated using the homologous series of n-alkanes C_8_–C_20_, Sigma-Aldrich (St. Louis, MO, USA). The identification of the constituents of the EOs and mixtures was performed by comparing the mass spectra and retention indices (RI) with those of existing standard substances in the library [37].

### 3.5. Preliminary Toxicity Assay

Toxicity tests of *Piper* essential oils (EOs) and mixtures were performed against larvae of the microcrustacean *Artemia salina*, as described in [38,39]. *A. salina* eggs were incubated at room temperature (25 °C) in an aquarium containing artificial salt water (brine) composed of a mixture of 46 g of NaCl, 22 g of MgCl_2_.6H_2_O, 8 g of Na_2_SO_4_, 2.6 g of CaCl_2_.6H_2_O, and 1.4 g of KCl in 2 L of deionized water. The pH was adjusted to 8–9 using sodium carbonate (Na_2_CO_3_). Twenty-four hours after the eggs hatched, solutions of the EOs and their mixtures were prepared at concentrations of 1, 5, 10, 25, and 50 µg/mL, as determined from preliminary tests, and at 100 µg/mL, using brine and 5% dimethyl sulfoxide (DMSO) as diluents. Ten *A. salina* larvae were added to each tube containing the solutions, and after 24 h, the mortality rate of the larvae was measured. The assays were performed in triplicate (*r* = 3), and preliminary toxicity was assessed based on the median lethal concentration (LC_50_).

### 3.6. Larvicidal Assay

The larvicidal activity of *Piper* essential oils (EOs) and mixtures was evaluated against third-stage *Aedes aegypti* mosquito larvae following the methodology described by the World Health Organization [40], with adaptations. The hatching and larval rearing procedures were performed according to the methodology adapted from [41].

To perform the test, the larvae were immersed in 20 mL of EO solutions and mixtures at concentrations ranging from 10 to 250 μg/mL, determined from preliminary tests, using polysorbate 80 as an emulsifier. After 24 h of exposure at room temperature (25 °C), larval mortality was measured. The experiments were performed in quintuplicate (*r* = 5) with ten larvae (*n* = 10) for each replicate, and a negative control was evaluated using the same procedure without adding a sample. The larvicidal activity of the EOs and mixtures was evaluated in terms of their average lethal concentrations (LC_50_ and LC_90_), according to the classification proposed by [4,30,31] regarding their action against *A. aegypti* larvae: LC_50_ ≤ 50 µg/mL (highly active); 50 < LC_50_ ≤ 100 µg/mL (active); 100 < LC_50_ ≤ 750 µg/mL (effective); and LC_50_ > 750 µg/mL (inactive).

### 3.7. Statistical Analysis

Mortality data from preliminary toxicity and larvicidal assays were pooled and presented as mean ± standard deviation. The concentrations required to cause 50 and 90% mortality of the tested larval population (LC_50_ and LC_90_, respectively) were calculated by nonlinear regression analysis using GraphPad Prism 10 software. A one-way ANOVA was performed, followed by Tukey’s post hoc test, to assess statistical differences among the analyzed samples. Differences were considered statistically significant at *p* < 0.05.

### 3.8. Molecular Docking Procedure

Molecular docking simulations were performed via the Auto Dock 4.2 program package [42]. The crystal structure of ‘acetylcholinesterase’ (pdb: 4m0e) was procured from the RSCB protein data bank (https://www.rcsb.org/ (accessed on 4 March 2026)) [43]. First, water molecules were removed, and then missing hydrogens and Gasteiger charges were added to the system during the preparation of the receptor input file. Then, AutoDock Tools (2010 version) was used to prepare the ligand and protein files (PDBQT). Pre-calculation of grid maps was performed with AutoGrid to save time during docking. Geometric optimization of all compounds was performed using ACD (3D viewer) software (2017 version) (http://www.filefacts.com/acd3d-viewer-freeware-info (accessed on 4 March 2026)), and the visualization and analysis of interactions were performed using Discovery Studio 2017R2 and PyMOL 0.99rc6 [44].

### 3.9. ADME Properties

The pharmacokinetic and drug-likeness properties of the selected phytocompounds were evaluated using ADME (absorption, distribution, metabolism, and excretion) descriptors via a SwissADME online server (http://www.swissadme.ch/ (accessed on 4 March 2026)).

## 4. Conclusions

The essential oils of *Piper callosum*, *Piper divaricatum*, and *Piper aduncum*, as well as their binary and ternary mixtures, showed chemical profiles rich in bioactive constituents, with a predominance of phenylpropanoids such as safrole, dillapiole, eugenol, eugenol acetate, and methyl eugenol. The abundance of these compounds appears to be closely associated with the larvicidal activity observed against *Aedes aegypti* larvae, reinforcing the relevance of these metabolites as potential natural agents for vector control. Among the evaluated oils, *P. aduncum* demonstrated the highest larvicidal effectiveness, followed by *P. callosum*, while *P. divaricatum* showed comparatively lower activity. The mixtures exhibited variable biological responses, indicating that the interaction among essential oil constituents can modulate activity through synergistic or antagonistic effects. In particular, the combination of *P. divaricatum* and *P. aduncum* showed a synergistic effect, maintaining strong larvicidal performance while preserving the major compounds of the species involved. An additional important finding was the reduction in toxicity observed for the mixtures against *Artemia salina* when compared to the individual oils, suggesting that combined formulations may offer a safer alternative for practical application. These results indicate that the essential oils of the studied species and their combinations represent promising larvicidal agents for the control of *A. aegypti*, contributing to the development of effective and environmentally safer strategies for mosquito management. The in silico investigation supported the experimental results by demonstrating favorable interactions between the major phytoconstituents and acetylcholinesterase, an important enzymatic target in insect neurophysiology, suggesting that enzyme inhibition may be one of the mechanisms involved in larvicidal activity. Furthermore, ADME predictions indicated favorable pharmacokinetic behavior and drug-likeness profiles, reinforcing the biological plausibility of the proposed mechanism of action. Future studies should focus on the isolation and individual evaluation of the major phytoconstituents, as well as their synthetic analogues, to better clarify their specific contributions to larvicidal activity and toxicity. Additional validation through enzymatic assays and target-specific bioassays will also be essential to confirm the molecular mechanisms proposed by the in silico analysis and to fill potential gaps regarding the synergistic and antagonistic interactions observed among the essential oil mixtures.

## Figures and Tables

**Table 1:** Chemical composition of the EOs of *P. callosum*, *P. divaricatum*, *P. aduncum*, and mixtures of these oils. Each species is represented by the first letters of genus and species (e.g., *Piper callosum* = *Pc*), and the compounds are arranged in ascending order of retention index (RI).

RI^L^	RI^C^	Compound	Concentration (%)						
			*Pc*	*Pd*	*Pa*	*PcPd*	*PcPa*	*PdPa*	*PcPdPa*
932	932	α-pinene	1.50	-	0.21	0.67	0.83	-	0.50
969	970	sabinene	0.45	-	-	-	-	-	0.07
974	977	**β-pinene**	**8.85**	-	0.56	3.41	3.80	-	2.33
988	987	myrcene	0.99	-	-	-	-	-	-
1014	1016	α-terpinene	0.64	-	0.16	-	0.32	-	0.25
1022	1022	o-cymene	-	-	0.14	-	-	-	-
1025	1029	β-phellandrene	-	-	0.80	-	-	-	-
1025	1027	limonene	0.24	-	-	-	0.32	0.28	0.25
1026	1030	1,8-cineol	2.21	-	-	0.40	0.69	-	0.51
1032	1032	(Z)-β-ocimene	-	-	2.33	-	0.97	0.91	0.84
1044	1043	**(** ***E*** **)-β-ocimene**	-	3.67	**6.20**	1.27	2.43	3.95	2.74
1054	1056	γ-terpinene	3.45	-	1.32	1.22	1.92	0.39	1.30
1086	1084	terpinolene	0.51	-	0.45	-	-	-	0.26
1174	1178	terpinen-4-ol	-	-	2.23	-	0.67	0.74	0.49
1249	1250	piperitone	-	-	4.25	-	1.86	1.84	1.22
1285	1286	**safrole**	**59.42**	6.04	-	**33.40**	**32.41**	3.76	**19.75**
1335	1330	δ-elemenol	-	1.21	0.69	0.42	-	0.75	0.56
1356	1352	**eugenol**	-	**18.26**	-	**12.11**	-	**12.87**	**8.34**
1374	1373	α-copaene	0.62	-	0.22	0.29	0.32	0.13	0.31
1389	1388	**β-elemene**	-	**12.96**	0.68	**7.45**	-	**6.29**	**5.13**
1403	1397	**methyl eugenol**	**12.56**	**12.60**	-	**13.63**	**6.50**	**7.88**	**8.40**
1417	1416	**(** ***E*** **)-caryophyllene**	0.54	**9.57**	3.22	**5.26**	1.66	**5.24**	**4.36**
1430	1427	β-copaene	-	0.41	0.58	-	-	0.38	0.27
1452	1452	α-humulene	-	0.77	0.89	0.29	0.31	0.71	0.51
1471	1472	dauca-5,8-diene	0.67	-	-	0.30	0.18	-	0.27
1478	1477	**γ-muurolene**	1.16	**8.75**	3.95	4.71	2.27	4.97	4.28
1489	1485	β-selinene	-	0.12	-	-	-	0.14	-
1493	1488	trans-Muurola-4(14),5-diene	-	-	0.21	-	-	-	-
1500	1492	**bicyclogermacrene**	-	**5.12**	2.96	2.36	1.21	3.50	2.49
1500	1495	α-muurolene	-	0.20	0.15	-	-	0.14	0.09
1508	1503	germacrene A	-	0.37	-	-	-	-	-
1513	1509	γ-cadinene	-	-	0.16	-	-	-	-
1517	1515	myristicin	-	-	4.42	-	2.12	-	1.68
1521	1513	**eugenol acetate**	-	**18.02**	-	**9.47**	-	**8.50**	**6.52**
1522	1515	δ-cadinene	0.39	0.28	-	0.33	-	2.35	-
1555	1542	elemicin	3.72	-	-	1.65	1.53	0.13	1.21
1559	1554	germacrene B	-	-	0.11	-	-	-	-
1561	1557	E-nerolidol	-	0.19	-	-	-	0.17	0.08
1577	1572	spatulenol	-	1.15	0.89	0.61	0.22	1.21	0.80
1582	1581	caryophyllene oxide	-	0.13	0.23	-	-	0.18	-
1592	1589	viridiflorol	-	-	1.41	-	0.33	0.60	0.34
1620	1624	**dillapiole**	-	-	**55.92**	-	**35.22**	**29.51**	**22.06**
1638	1640	epi-α-cadinol (tau-cadinol)	-	-	0.34	-	-	0.22	-
1644	1642	α-muurolol (Torreyol)	0.36	-	0.45	-	-	-	0.16
1652	1650	α-eudesmol	1.41	-	-	0.75	0.71	-	-
1652	1652	α-cadinol	-	-	0.45	-	-	-	-
1677	1668	apiol	-	-	0.27	-	-	0.08	-
Monoterpene hydrocarbons			16.63	3.67	12.17	6.57	10.59	5.53	8.54
Oxygenated monoterpenes			2.21	0.00	6.48	0.04	3.22	2.58	2.22
Sesquiterpene hydrocarbons			3.38	39.76	13.82	21.41	5.95	24.60	18.27
Oxygenated sesquiterpenes			1.77	1.47	3.77	1.36	1.26	2.38	1.38
Phenylpropanoids			75.50	54.92	60.61	70.26	77.78	62.73	67.96
Total			99.69	99.82	96.85	100.00	98.80	97.82	98.37

Legend: RI^L^ = Adams literature retention index; RI^C^ = calculated retention index; *Pc* = *Piper callosum*; *Pd* = *Piper divaricatum*; *Pa* = *Piper aduncum*; *PcPd* = mixture of *P. callosum*/*P. divaricatum* essential oils; *PcPa* = mixture of EOs from *P. callosum*/*P. aduncum*; *PdPa* = mixture of EOs from *P. divaricatum*/*P. aduncum;* and *PcPdPa* = mixture of EOs from *P. callosum*/*P. divaricatum*/*P. aduncum.* Major compounds (concentration ≥ 5%) are in bold.

**Table 2:** Mortality data of *Piper* OEs and mixtures against larvae of the microcrustacean *A. salina* after 24 h of exposure.

Essential Oil/Mixture	Concentration (µg/mL)	Mortality (%)
*Pc*	1	0.0 ± 0.0
	5	16.7 ± 4.4
	10	33.3 ± 4.4
	25	43.3 ± 4.4
	50	100.0 ± 0.0
	100	100.0 ± 0.0
*Pd*	1	0.0 ± 0.0
	5	6.7 ± 4.4
	10	10.0 ± 0.0
	25	40.0 ± 6.7
	50	63.3 ± 8.9
	100	100.0 ± 0.0
*Pa*	1	0.0 ± 0.0
	5	10.0 ± 6.7
	10	13.3 ± 4.4
	25	23.3 ± 4.4
	50	86.7 ± 4.4
	100	100.0 ± 0.0
*PcPd*	1	0.0 ± 0.0
	5	0.0 ± 0.0
	10	0.0 ± 0.0
	25	70.0 ± 6.7
	50	100.0 ± 0.0
	100	100.0 ± 0.0
*PcPa*	1	0.0 ± 0.0
	5	0.0 ± 0.0
	10	10.0 ± 0.0
	25	33.3 ± 4.4
	50	80.0 ± 6.7
	100	100.0 ± 0.0
*PdPa*	1	0.0 ± 0.0
	5	0.0 ± 0.0
	10	0.0 ± 0.0
	25	23.3 ± 4.4
	50	86.7 ± 4.4
	100	100.0 ± 0.0
*PcPdPa*	1	0.0 ± 0.0
	5	0.0 ± 0.0
	10	13.3 ± 4.4
	25	53.3 ± 4.4
	50	93.3 ± 4.4
	100	100.0 ± 0.0

Legend: *Pc* = *Piper callosum*; *Pd* = *Piper divaricatum*; *Pa* = *Piper aduncum*; *PcPd* = mixture of OEs from *P. callosum*/*P. divaricatum*; *PcPa* = mixture of EOs from *P. callosum*/*P. aduncum*; *PdPa* = mixture of EOs from *P. divaricatum*/*P. aduncum*; and *PcPdPa* = mixture of EOs from *P. callosum*/*P. divaricatum*/*P. aduncum*.

**Table 3:** Mortality data of *Piper* EOs and mixtures against *A. aegypti* larvae after 24 h of exposure.

Essential Oil/Mixture	Concentration (µg/mL)	Mortality (%)
*Pc*	10	0.0 ± 0.0
	25	2.0 ± 4.5
	50	14.0 ± 11.4
	75	96.0 ± 8.9
	100	100.0 ± 0.0
*Pd*	50	4.0 ± 5.5
	100	22.0 ± 8.4
	150	76.0 ± 5.5
	200	98.0 ± 4.5
	250	100.0 ± 0.0
*Pa*	10	4.0 ± 5.5
	25	30.0 ± 7.1
	50	98.0 ± 4.5
	75	100.0 ± 0.0
	100	100.0 ± 0.0
*PcPd*	50	0.0 ± 0.0
	100	66.0 ± 5.5
	150	96.0 ± 5.5
	200	98.0 ± 4.5
	250	100.0 ± 0.0
*PcPa*	10	0.0 ± 0.0
	25	2.0 ± 4.5
	50	10.0 ± 10.0
	75	98.0 ± 4.5
	100	100.0 ± 0.0
*PdPa*	10	4.0 ± 5.5
	25	2.0 ± 4.5
	50	14.0 ± 5.5
	75	98.0 ± 4.5
	100	100.0 ± 0.0
*PcPdPa*	10	0.0 ± 0.0
	25	0.0 ± 0.0
	50	10.0 ± 12.2
	75	100.0 ± 0.0
	100	100.0 ± 0.0

Legend: *Pc* = *Piper callosum*; *Pd* = *Piper divaricatum*; *Pa* = *Piper aduncum*; *PcPd* = mixture of OEs from *P. callosum*/*P. divaricatum*; *PcPa* = mixture of EOs from *P. callosum*/*P. aduncum*; *PdPa* = mixture of EOs from *P. divaricatum*/*P. aduncum*; and *PcPdPa* = mixture of EOs from *P. callosum*/*P. divaricatum*/*P. aduncum*.

**Table 4:** Binding energy of the docked compounds in the binding cavity of acetylcholinesterase (pdb: 4m0e).

Compound	Binding Energy (kcal/mol)
β-pinene	−5.1
(*E*)-β-ocimene	−6.3
safrole	−6.7
eugenol	−6.2
β-elemene	−6.8
methyl eugenol	−6.2
(*E*)-caryophyllene	−6.9
γ-muurolene	−7.2
bicyclogermacrene	−6.6
eugenol acetate	−6.5
dillapiole	−6.6
co-crystallized ligand	−8.3

**Table 5:** In silico ADME outputs of the tested phytocompounds.

Entry	a	b	c	d	e	f	g	h	i	j	k
GI absorption *	Low	Low	High	High	Low	High	Low	Low	Low	High	High
BBB permanent *	Yes	Yes	Yes	Yes	No	Yes	No	No	No	Yes	Yes
P–gp substrate *	No	No	No	No	No	No	No	No	No	No	No
CYP1A2 inhibitor *	No	No	Yes	Yes	No	Yes	No	No	No	Yes	Yes
CYP2C19 inhibitor *	No	No	No	No	Yes	No	Yes	Yes	Yes	No	No
CYP2C9 inhibitor *	Yes	No	No	No	Yes	No	Yes	Yes	Yes	No	No
CYP2D6 inhibitor *	No	No	No	No	No	No	No	No	No	No	No
CYP3A4 inhibitor *	No	No	No	No	No	No	No	No	No	No	No
Log Kp (cm/s) ^A^*	−4.18	−4.11	−5.19	−5.69	−3.21	−5.60	−4.44	−4.49	−4.61	−5.93	−5.70
Lipinski **	Yes	Yes	Yes	Yes	Yes	Yes	Yes	Yes	Yes	Yes	Yes
Veber **	Yes	Yes	Yes	Yes	Yes	Yes	Yes	Yes	Yes	Yes	Yes
Egan **	Yes	Yes	Yes	Yes	Yes	Yes	Yes	Yes	Yes	Yes	Yes
Bioavailability Score **	0.55	0.55	0.55	0.55	0.55	0.55	0.55	0.55	0.55	0.55	0.55
TPSA (Å^2^) ***	00.00	00.00	18.46	29.46	00.00	18.46	00.00	00.00	00.00	35.53	36.92
Consensus Log Po/*w* ****	3.42	3.40	2.52	2.25	4.65	2.58	4.24	4.17	4.13	2.55	2.43

Legend: ^A^: skin permeability, Pharmacokinetics *, Drug-likeness **, Physicochemical properties ***, and Lipophilicity ****, a: β-pinene, b: (*E*)-β-ocimene, c: safrole, d: eugenol, e: β-elemene, f: methyl eugenol, g: (*E*)-caryophyllene, h: γ-muurolene, i: bicyclogermacrene, j: eugenol acetate, and k: dillapiole.

## Data Availability

The original contributions presented in this study are included in the article. Further inquiries can be directed to the corresponding author.
